# Clinical Features and Treatment Modes of Mandibular Fracture at the Department of Oral and Maxillofacial Surgery, Shimane University Hospital, Japan

**DOI:** 10.1371/journal.pone.0136278

**Published:** 2015-09-03

**Authors:** Hiroto Tatsumi, Eiji Nakatani, Takahiro Kanno, Yoshiki Nariai, Tatsuo Kagimura, Joji Sekine

**Affiliations:** 1 Department of Oral and Maxillofacial Surgery, Shimane University Faculty of Medicine, Izumo, Japan; 2 Translational Research Informatics Center, Foundation Biomedical Research and Innovation, Kobe, Japan; Medical University of South Carolina, UNITED STATES

## Abstract

**Background:**

The number of elderly patients with maxillofacial trauma is rapidly increasing due to active lifestyles and longevity. Shimane prefecture has the fastest growing proportion of elderly individuals in Japan. The aim of this study was to reveal the distinctive features and treatment modes of mandibular fracture treatment mode in patients requiring hospitalization at the Department of Oral and Maxillofacial Surgery, Shimane University Hospital, Japan.

**Patients and Methods:**

Patient age, sex, period between injury and first consultation, years since injury, cause of injury, fracture site, treatment, and duration of hospitalization were evaluated. Univariate Poisson regression, relative risk with 95% confidence interval based on the Wald test, Fisher’s exact test, and Kruskal-Wallis test were used to explore associations among clinical and demographic variables.

**Results:**

In total, 305 patients were diagnosed with and hospitalized for mandibular fracture from 1980 to 2010. Younger age increased the risk for mandibular fracture. Incidence was higher in males than females, particularly in the young, but the male to female ratio decreased with age. The period until first hospital consultation decreased progressively over the study period. Fall was a much more frequent cause in patients aged ≥60 than in those aged <60 years. Mandibular fracture with condyle, symphysis, and angle involvement were most common and were associated with sex, age, and treatment mode. Length of hospitalization has decreased since 1980.

**Conclusion:**

In our department, patients aged ≥60 years accounted for a greater proportion of mandibular fracture cases than in many previous studies, reflecting the greater proportion of elderly residents in Shimane prefecture.

## Introduction

The incidence of maxillofacial fracture varies with population density, living environment, socioeconomic status, and road traffic conditions [1–5]. Most cases of maxillofacial trauma involve mandibular fracture [2–4], which in Japan is usually managed by departments of oral and maxillofacial surgery [6].

The number of elderly patients with maxillofacial trauma has increased in recent decades because of changes in lifestyle and an increase in the proportion of elderly patients in the population [1]. Statistics have shown that 22.7% of the Japanese population is ≥65 years of age, with elderly individuals accounting for a growing portion of the Japanese population. Indeed, the proportion of elderly is expected to expand by an additional 41.8% by the year 2050 [7]. Likewise, in other developed countries, the proportion of individuals ≥65 years of age is expected to increase to 26.2% [8]. With demographic and various social changes, such as the greater number of elderly living alone and leading an active retirement, the elderly population may be at increased risk for trauma, including maxillofacial fracture.

Shimane prefecture, located in western Japan, has the most rapidly increasing proportion of elderly individuals in Japan. Among its population of 710,000, approximately 31.1% are elderly [7]. Therefore, this area is a suitable setting for studying epidemiological changes associated with a rapidly aging society.

The aim of this study was to reveal the distinctive features of mandibular fracture cases hospitalized at the Department of Oral and Maxillofacial Surgery, Shimane University Hospital located in the center of the Shimane prefecture, and to elucidate the clinical features and treatment modes of mandibular fracture.

## Method

### Patients

Shimane University Hospital maintains a database of all patients diagnosed as having and hospitalized for mandibular fracture between April 1980 and March 2010. In this study, patients who did not require hospital treatment or had minor injuries were excluded.

This study was conducted in accordance with the Declaration of Helsinki, and all patient data were unlinked and anonymous. The unlinked anonymity was ensured by the president of Shimane University Faculty of Medicine.

### Evaluated variables

Patient age, sex, period between injury and first hospital consultation, years since injury, cause of injury, fracture site, treatment mode, and length of hospitalization were evaluated.

### Statistical analysis

To identify factors associated with the incidence of mandibular fracture, univariate Poisson regression model was performed. The relative risk (RR), the 95% confidence interval (CI) calculated by the Wald test were determined and a likelihood ratio test was conducted for each factor. Fisher’s exact test or Cochran-Armitage test for trend was performed to identify categorical variables associated with age and the Kruskal-Wallis test was used to identify categorical variables associated with period from fracture to first hospital consultation and length of hospitalization. Time periods are expressed as the mean and standard deviation (SD). Here, p<0.05 was considered significant. All statistical analyses were performed using SAS version 9.3 (Cary, NC).

## Results

### Incidence of mandibular fracture

Between April 1980 and March 2010, 305 patients were diagnosed with and hospitalized for mandibular fracture at Shimane University Hospital. Both sex (p<0.001) and age (p<0.001) were associated with the incidence of mandibular fracture but decade of injury was not (p = 0.106; Table 1). The incidence of mandibular fracture was higher in males than females (RR: 2.51, 95% CI: 1.95−3.21; Table 1). Younger age increased the risk for mandibular fracture (Table 1).

**Table 1:** Univariate Poisson regression analysis of mandible fracture incidence in hospitalized patients from Shimane prefecture.

Variable	Category, n = 305	n	Relative risk	95% CI	Likelihood ratiotest
Decade	1980−1990	88	1.00		0.106
	1991−2000	118	1.34	1.02−1.77	
	2001−2010	99	1.13	0.84−1.50	
Sex	Female	87	1.00		<0.001
	Male	218	2.51	1.95−3.21	
Age, years	0−19	91	1.00		<0.001
	20−39	77	0.85	0.62−1.15	
	40−59	62	0.68	0.49−0.94	
	60−79	59	0.65	0.47−0.90	
	≥80	16	0.18	0.10−0.30	

CI: Confidence interval.

### Association between age and sex

The male to female ratio decreased with age (p<0.001, Table 2) from 5.50, 3.53, and 3.13, respectively, for the youngest groups (0–19, 20–39, 40–59 years) to 1.03 and 0.33 for the oldest two groups (60–79 and ≥80).

**Table 2:** Association between age and sex.

Variable	Category, n = 305	n	Age, n (%)					CochranArmitage test
			0–19 years,	20–39,	40–59,	60–79,	≥80,	
			n = 91	n = 77	n = 62	n = 59	n = 16	
Sex	Male	218	77 (84.6)	60 (77.9)	47 (75.8)	30 (50.8)	4 (25.0)	<0.001
	Female	87	14 (15.4)	17 (22.1)	15 (24.2)	29 (49.2)	12 (75.0)	

### Period from fracture to first hospital consultation

The median (range) and mean (SD) periods from fracture to first hospital consultation (days) were 1 (0−40) and 2.8 (4.7). Further, the decade was associated with the period from fracture to first hospital consultation (p = 0.004), while sex and age were not (p = 0.830 and p = 0.559, respectively; Table 3). The delay between injury and first hospital consultation decreased progressively with decade from 1980−1990 to 2001−2010 (Table 3).

**Table 3:** Factor associated with period from fracture to hospital consultation.

Variable	Category, n = 305	Period from fracture to hospital consultation, days		Kruskal-Wallis test
		n	Mean	
			(%)	
Decade of	1980–1990	88	4.3 (7.0)	0.004
injury	1991–2000	118	2.6 (3.5)	
	2001–2010	99	1.6 (2.5)	
Sex	Male	218	2.7 (4.9)	0.830
	Female	87	2.8 (4.1)	
Age at injury	0–19 years	91	2.4 (4.6)	0.599
	20–39	77	2.8 (5.1)	
	40–59	62	3.8 (5.6)	
	60–79	59	2.3 (3.2)	
	≥80	16	2.3 (2.7)	

### Cause of mandibular fracture

Both age and sex were associated with the cause of mandibular fracture (both p<0.001; Table 4). Fall was a more common cause in those aged ≥60 years than in those aged <60 years (chi-squared test: p<0.001; Table 4). Fall caused the majority of mandibular fractures in patients 70−79 years old and was the only cause reported in patients ≥80 years old. Traffic accidents were a more frequent cause in males than females for all age groups except for those aged ≥80 years (no cases). Males accounted for almost all sports accidents, and all cases (both male and female) were aged <40 years (Table 4). Males also accounted for the vast majority of work-related mandibular fractures, and almost 60% occurred in the 40−59 age group (Table 4). Finally, males accounted for most cases of violence-related mandibular fracture, almost all in males younger than 40 years. All violence-related cases, both male and female, were patients aged <60 years (Table 4).

**Table 4:** Causes of fracture stratified by sex and age.

Variable	Category, n = 305	n	Causes of fracture, n (%)						Fisher's exact test
			Fall, n = 113	Traffic accident, n = 91	Sports accident, n = 39	Work related, n = 30	Violence, n = 29	Others, n = 3	
Sex	Male	218	63 (55.8)	59 (64.8)	38 (97.4)	29 (96.7)	26 (89.7)	3 (100.0)	<0.001
	Female	87	50 (44.2)	32 (35.2)	1 (2.6)	1 (3.3)	3 (10.3)	0 (0.0)	
Age	0–19 years	91	23 (20.4)	29 (31.9)	27 (69.2)	3 (10.0)	9 (31.0)	0 (0.0)	<0.001
	20–39	77	16 (14.2)	26 (28.6)	12 (30.8)	6 (20.0)	16 (55.2)	1 (33.3)	
	40–59	62	20 (17.7)	20 (22.0)	0 (0.0)	17 (56.7)	4 (13.8)	1 (33.3)	
	60–79	59	38 (33.6)	16 (17.6)	0 (0.0)	4 (13.3)	0 (0.0)	1 (33.3)	
	≥80	16	16 (14.2)	0 (0.0)	0 (0.0)	0 (0.0)	0 (0.0)	0 (0.0)	

### Site of mandibular fracture

In the 305 patients hospitalized for mandible fracture, 274 (89.8%) had mandibular fracture only, while 25 cases (8.2%) were accompanied by maxilla fracture, 3 (1.0%) by zygoma fracture, and 3 (1.0%) by maxilla and zygoma fractures. In both sexes, the condyle was the most common fracture site (126 cases, 41.3%), followed by the symphysis (114, 37.4%), angle (82, 26.9%), body (41, 13.4%), alveolar process (28, 9.2%), and ramus (6, 2.0%). The facture site was associated with sex for condyle (p = 0.003), symphysis (p = 0.013), and angle (p = 0.022), but not for the body, alveolar process, or ramus (p = 0.710, p = 0.083, and p = 0.188, respectively; Table 5). The facture site was also associated with age for the condyle and angle (both p<0.001) and the symphysis (p = 0.001) but not for the ramus, body, or alveolar process (p = 0.818, 0.049, and p = 0.568, respectively; Table 6). Furthermore, fracture at the condyle, angle, and alveolar process was associated with surgical/non-surgical treatment (p = 0.047, p = 0.004, and p<0.001, respectively), while fracture of the ramus or body was not (p = 0.526 and p = 0.300, respectively; Table 7). Fractures sites in cases of multiple mandibular fractures are shown in Table 8.

**Table 5:** Site of mandible fracture stratified by sex.

Site of mandible fracture, n = 305	n	Sex, n (%)		Fisher's exact test
		Male, n = 218	Female, n = 87	
Condyle	126	78 (35.8)	48 (55.2)	0.003
Ramus	6	6 (2.8)	0 (0.0)	0.188
Angle	82	67 (30.7)	15 (17.2)	0.022
Body	41	28 (12.8)	13 (14.9)	0.710
Symphysis	114	91 (41.7)	23 (26.4)	0.013
Alveolar process	28	16 (7.3)	12 (13.8)	0.083

**Table 6:** Site of mandible fracture stratified by age.

Site of mandible fracture, n = 305	n	Age, n (%)					Cochran-Armitage test
		0–19 years, n = 91	20–39, n = 77	40–59, n = 62	60–79, n = 59	≥80, n = 16	
Condyle	126	26 (28.6)	26 (33.8)	29 (46.8)	35 (59.3)	10 (62.5)	<0.001
Ramus	6	1 (1.1)	2 (2.6)	3 (4.8)	0 (0.0)	0 (0.0)	0.818
Angle	82	32 (35.2)	28 (36.4)	12 (19.4)	9 (15.3)	1 (6.3)	<0.001
Body	41	8 (8.8)	11 (14.3)	7 (11.3)	11 (18.6)	4 (25.0)	0.049
Symphysis	114	42 (46.2)	31 (40.3)	27 (43.5)	11 (18.6)	3 (18.8)	0.001
Alveolar process	28	11 (12.1)	4 (5.2)	6 (9.7)	7 (11.9)	0 (0.0)	0.568

**Table 7:** Site of mandible fracture stratified by treatment.

		Treatment, n (%)		
Site of mandible fracture, n = 305	n	Surgical, n = 102	Non-surgical, n = 203	Fisher's exact test
Condyle	126	33 (32.4)	93 (45.8)	0.047
Ramus	6	3 (2.9)	3 (1.5)	0.526
Angle	82	17 (16.7)	65 (32)	0.004
Body	41	18 (17.6)	23 (11.3)	0.300
Symphysis	114	44 (43.1)	70 (34.5)	0.232
Alveolar process	28	23 (22.5)	5 (2.5)	<0.001

**Table 8:** Site of mandible fracture.

Site of mandible fracture, n = 305	n	%
Condyle	63	20.7
Condyle + ramus	1	0.3
Condyle + angle	9	3
Condyle + angle + symphysis	1	0.3
Condyle + body	11	3.6
Condyle + symphysis	40	13.1
Condyle + symphysis + alveolar process	1	0.3
Ramus + angle + symphysis	1	0.3
Ramus + symphysis	4	1.3
Angle	37	12.1
Angle + body	6	2
Angle + body + symphysis	1	0.3
Angle + symphysis	27	8.9
Body	14	4.6
Body + symphysis	9	3
Symphysis	28	9.2
Symphysis + alveolar process	2	0.7
Alveolar process	25	8.2
Unknown	25	8.2

### Treatment for mandibular fracture

The treatments provided are summarized in Table 9. The most common surgical procedure was insertion of a titanium plate. About two-thirds of patients were treated conservatively, mainly by intermaxillary fixation.

**Table 9:** Treatment for mandible fracture.

Treatment, n = 305		n	%
Surgical	Titanium plate	52	
	Teeth ligation	13	
	Absorbable plate	12	
	Other	12	
	Titanium plate and other	4	
	Kirschner wire	3	
	Bone depletion	1	
	(Missing)	5	
	total	102	33.4
Conservative	Intermaxillary fixation	146	
	Chin cap	24	
	Elastic vantage	23	
	total	193	63.3
Others	No treatment	8	
	(Missing)	2	
	total	10	3.3

### Factor associated with length of hospitalization

The median (min−max) and mean (SD) length of hospitalization (days) were 23 (3−88) and 26.0 (14.6). Both decade of injury and age were associated with length of hospitalization (both p<0.001), while sex and treatment with/without surgical were not (p = 0.452 and p = 0.124; Table 10). Length of hospitalization decreased progressively from 1980−1990 to 2001−2010 (Table 10). Length was longer in the 20−39 and 40−59 year age groups (Table 10).

**Table 10:** Factors associated with length of hospitalization.

Variable	Category, n = 305	Length of hospitalization, days		Kruskal-Wallis test
		n	Mean (standard deviation)	
Decade	1980–1990	88	31.5 (15.8)	<0.001
	1991–2000	118	27.1 (14.3)	
	2001–2010	99	19.8 (11.4)	
Sex	Male	218	25.6 (15.4)	0.452
	Female	87	26.2 (14.3)	
Age,	0−19 years	91	21.7 (11.5)	<0.001
years	20−39	77	28.4 (15.7)	
	40−59	62	32.2 (16.5)	
	60−79	59	24.7 (13.7)	
	≥80	16	19.8 (11.3)	
Therapy	Surgical	102	28.2 (16.1)	0.124
	Non-surgical	193	24.9 (13.8)	

## Discussion

This study revealed that incidence of mandibular fracture was highest in younger patients, especially in males, in accord with previous studies [9–12]. However, unlike previous reports, the number of the patients aged ≥60 years in our study population was strikingly high at 24.6%, compared to only 3.0% and 6.3% in studies of other regions [13, 14]. Moreover, several previous studies reported that only 3.2%−10.0% of patients with mandibular fracture were ≥50 years old [9–11], again substantially lower than in our department. Thus, the epidemiology of mandibular fracture is expected to differ among regions, depending on demographic make-up (percentage of elderly), and may be more common in aging populations than indicated in previous studies.

Mandibular fracture occurs more frequently in males [10–17], with male to female ratios ranging from 2.3:1 to 7.4:1 [15, 17], so the ratio found in our department for the entire patient group (2.5:1) falls within this range, albeit on the lower end. Other studies have reported lower ratios in old patients, such as 1.1:1 in patients aged ≥60 years [1], comparable to the 0.8:1 calculated here. Thus, the ratio of male to female patients treated is expected to change depending on the proportion of elderly residents in the population. Indeed, the oldest patients treated in Shimane prefecture were female (Table 2) [7].

The period until first hospital consultation has decreased in our department over the study period, possibly due to the development of more accessible transportation and establishment of an emergency system. In many studies, the most common cause of mandibular fracture was traffic accidents [10–12, 14, 16, 18], although some studies have reported assault or other forms of violence to be the most common cause [9, 13, 17]. In our department, the most common cause of fractures was fall (n = 113), and in almost half of all cases (n = 54). However, among patients aged <60 years, traffic accidents (n = 75) was the single most common cause. Similarly, a previous study [1] reported that falls was the most common cause (43.5%) in patients aged ≥60 years. Moreover, another study [19] reported that facial injuries caused by falls were more common among elderly women with limited mobility and osteoporosis.

The most common site of mandibular fractures manifested in our department was the condyle, followed by the symphysis and angle, and fractures at these locations were associated with age. In general, the most common sites of mandibular fracture are the condyle and angle (especially in the presence of an impacted or semi-erupted third molar), mental foramen or body, parasymphysis, and any part of the dental alveolus [20]. However, the most common fracture site in patients aged ≥60 years in this study group was the symphysis, followed by the body. Although the reason for this is unclear, it is possible that elderly patients with limited mobility, especially those living alone, can easily stumble and fall, striking their mandible against the ground directly without first falling into outstretched hands or arms, causing an indirect fracture of the condyle. In addition, it is also possible that dentition is associated with mandibular fractures in the elderly because they are often fully or partially edentulous, with atrophy of the jaw and osteoporosis [19].

In this study, fracture with condylar, angle, or alveolar involvement was also associated with treatment with/without surgery. More than half of all patients were treated with intermaxillary fixation, chin cap, elastic bandage, or other conservative treatment. In our department, conservative treatment is usually applied first, especially for condylar fractures. Our previous studies [21, 22] revealed that mandibular condylar fracture healing was delayed by aging, and thus a conservative treatment approach is more feasible for younger patients. However, a number of surgical procedures for mandibular condylar fractures have been reported using less invasive endoscopy [23], fixation by a mini-plate [24], and the retromandibular approach [25].

In our department, we also apply conservative treatment for mandibular angle fracture. As this fracture is most common in younger patients with healthy teeth, intermaxillary fixation is usually indicated. However, bony synthesis can take a long time, so surgical treatment methods are becoming more common in our department. On the other hand, mandibular alveolar fracture was treated surgically because it requires teeth ligation with reduction of deviated alveolar ridge.

In recent years, surgical approaches using computer-assisted navigation have been developed [26], and CAD/CAM machines can instantly produce plates [27, 28]. As more elderly patients require treatment for mandibular fracture, we must determine how best to treat this group.

## Conclusions

In our department, patients aged ≥60 years accounted for a greater proportion of mandibular fracture cases than in many previous studies, reflecting the greater proportion of elderly residents in Shimane prefecture.

## Supporting Information

S1 Checklist Our paper adheres to the STROBE guidelines for the reporting of observational studies in epidemiology. We completed STROBE checklist as an additional file.
(DOC)
