# Supplementary material for: Clinical Features and Treatment Modes of Mandibular Fracture at the Department of Oral and Maxillofacial Surgery, Shimane University Hospital, Japan
Source: PLoS One. 2015 Sep 3;10(9):e0136278. doi: 10.1371/journal.pone.0136278 (PMC4559417; doi:10.1371/journal.pone.0136278)
Supplement: S1 Checklist — We completed STROBE checklist as an additional file. (DOC) [file pone.0136278.s001.doc]

STROBE Statement—checklist of items that should be included in reports of observational studies

|  | Item No. | Recommendation | Page  No. | Relevant text from manuscript |
| --- | --- | --- | --- | --- |
| **Title and abstract** | 1 | (*a*) Indicate the study’s design with a commonly used term in the title or the abstract | 1 | Clinical characteristics and treatment of mandible fractures |
|  |  | (*b*) Provide in the abstract an informative and balanced summary of what was done and what was found | 4 | Compared to epidemiological findings reported previously in other countries |
| Introduction | | | |  |
| Background/rationale | 2 | Explain the scientific background and rationale for the investigation being reported | 6 | This area is a suitable setting for studying epidemiological changes associated with a rapidly aging society and the potential demands that maxillofacial surgery departments will need to address in the future as the population ages. |
| Objectives | 3 | State specific objectives, including any prespecified hypotheses | 6 | The aim of this study was to analyze the clinical features of and treatment strategies for mandible fractures focus on each generations treated |
| Methods | | | |  |
| Study design | 4 | Present key elements of study design early in the paper | 8 | diagnosed with and hospitalized for mandible fractures |
| Setting | 5 | Describe the setting, locations, and relevant dates, including periods of recruitment, exposure, follow-up, and data collection | 8 | In database of Shimane University Hospital, the patients had been diagnosed with and hospitalized for mandible fractures between April 1980 and March 2010 at were enrolled in this study. |
| Participants | 6 | (*a*) *Cohort study*—Give the eligibility criteria, and the sources and methods of selection of participants. Describe methods of follow-up  *Case-control study*—Give the eligibility criteria, and the sources and methods of case ascertainment and control selection. Give the rationale for the choice of cases and controls  *Cross-sectional study*—Give the eligibility criteria, and the sources and methods of selection of participants | 8 | diagnosed with and hospitalized for mandible fractures between April 1980 and March 2010 |
|  |  | (*b*) *Cohort study*—For matched studies, give matching criteria and number of exposed and unexposed  *Case-control study*—For matched studies, give matching criteria and the number of controls per case | 8 | Patients who did not require hospital treatment or minor injuries were excluded. |
| Variables | 7 | Clearly define all outcomes, exposures, predictors, potential confounders, and effect modifiers. Give diagnostic criteria, if applicable | 8 | diagnosed with and hospitalized for mandible fractures between April 1980 and March 2010. Patients who did not require hospital treatment or minor injuries were excluded. |
| Data sources/ measurement | 8* | For each variable of interest, give sources of data and details of methods of assessment (measurement). Describe comparability of assessment methods if there is more than one group | 8, 9 | Patient age, gender, period between injury and first consultation, years of injury, cause of injury, fracture site, treatment and duration of hospitalization were evaluated |
| Bias | 9 | Describe any efforts to address potential sources of bias | 8 | Patients who did not require hospital treatment or minor injuries were excluded. |
| Study size | 10 | Explain how the study size was arrived at | 10 | 305 patients |

Continued on next page

| Quantitative variables | 11 | Explain how quantitative variables were handled in the analyses. If applicable, describe which groupings were chosen and why |  | nothing |
| --- | --- | --- | --- | --- |
| Statistical methods | 12 | (*a*) Describe all statistical methods, including those used to control for confounding | 8, 9 | To explore factors associated with the incidence of mandible fracture, univariate Poisson regression model was performed. The relative risk (RR), the 95% confidence interval (CI) based on Wald test was calculated and likelihood ratio test in each factor was performed. |
|  |  | (*b*) Describe any methods used to examine subgroups and interactions | 8, 9 | To explore factor (of categorical variable) associated with age categories, Fisher’s exact test was performed. To explore factor (of categorical variable) associated with period from fracture to hospital consultation and hospitalization, Kruskal-Wallis test was performed. The period was summarized by mean and standard deviation (SD). |
|  |  | (*c*) Explain how missing data were addressed |  | nothing |
|  |  | (*d*) *Cohort study*—If applicable, explain how loss to follow-up was addressed  *Case-control study*—If applicable, explain how matching of cases and controls was addressed  *Cross-sectional study*—If applicable, describe analytical methods taking account of sampling strategy |  | nothing |
|  |  | (*e*) Describe any sensitivity analyses |  | nothing |
| Results | | | | |
| Participants | 13* | (a) Report numbers of individuals at each stage of study—eg numbers potentially eligible, examined for eligibility, confirmed eligible, included in the study, completing follow-up, and analysed | 10 | Between April 1980 and March 2010 at Shimane University Hospital, 305 patients had diagnosed with and hospitalized for mandible fracture. |
|  |  | (b) Give reasons for non-participation at each stage |  | nothing |
|  |  | (c) Consider use of a flow diagram |  | nothing |
| Descriptive data | 14* | (a) Give characteristics of study participants (eg demographic, clinical, social) and information on exposures and potential confounders | 10 | The factors associated with the incidence of mandible fracture were gender and age, but the year was not. The Incidence of mandible fracture for men was higher than that for women. The younger age was increased the risk of mandible fracture. |
|  |  | (b) Indicate number of participants with missing data for each variable of interest |  | nothing |
|  |  | (c) *Cohort study*—Summarise follow-up time (eg, average and total amount) | 13 | The median (min-max) and mean (SD) for period of hospitalization (days) were 23 (3-88) and 26.0 (14.6). |
| Outcome data | 15* | *Cohort study*—Report numbers of outcome events or summary measures over time | *10* | Between April 1980 and March 2010 at Shimane University Hospital, 305 patients had diagnosed with and hospitalized for mandible fracture. |
|  |  | *Case-control study—*Report numbers in each exposure category, or summary measures of exposure |  |  |
|  |  | *Cross-sectional study—*Report numbers of outcome events or summary measures |  |  |
| Main results | 16 | (*a*) Give unadjusted estimates and, if applicable, confounder-adjusted estimates and their precision (eg, 95% confidence interval). Make clear which confounders were adjusted for and why they were included |  | nothing |
|  |  | (*b*) Report category boundaries when continuous variables were categorized |  | nothing |
|  |  | (*c*) If relevant, consider translating estimates of relative risk into absolute risk for a meaningful time period |  | nothing |

Continued on next page

| Other analyses | 17 | Report other analyses done—eg analyses of subgroups and interactions, and sensitivity analyses | 12 | The mandible fracture with condyle, symphysis and angle associated with gender. The mandible fracture with condyle, symphysis and angle associated with age. The fracture with condyle, angle and alveolar associated with treatment with/without surgical. |
| --- | --- | --- | --- | --- |
| Discussion | | | | |
| Key results | 18 | Summarise key results with reference to study objectives | 18 | Mandibular fracture in shimane prefecture has been shown characteristics features in their etiology, patterns, and treatment modalities. |
| Limitations | 19 | Discuss limitations of the study, taking into account sources of potential bias or imprecision. Discuss both direction and magnitude of any potential bias | 18 | In this study, all patients showed no clear complications during the hospitalization periods. However, patients were not performed to analyze for complications of post-hospitalization or long-term period. In addition, this study did not evaluate the period hospitalization or age distribution the difference between surgical and non-surgical treatment. |
| Interpretation | 20 | Give a cautious overall interpretation of results considering objectives, limitations, multiplicity of analyses, results from similar studies, and other relevant evidence | 14, 15 | This concurs with other studies, which have reported the 21-30 age group to be the group that most frequently presents with mandibular fractures.  compared to only 3.0 and 6.3 % in other studies. In addition, several studies revealed that rate of patients aged ≥60 years were to range from 3.2 to 10.0.  The male-to-female ratio is reported to range from 2.3:1 to 7.4:1.  The most common cause of mandibular fracture is reported to be traffic accidents, although some investigators have reported assault or violence to be the most common causes. |
| Generalisability | 21 | Discuss the generalisability (external validity) of the study results | 18 | mandibular fracture in shimane prefecture has been shown characteristics features in their etiology, patterns, and treatment modalities. |
| Other information | |  | | |
| Funding | 22 | Give the source of funding and the role of the funders for the present study and, if applicable, for the original study on which the present article is based |  | nothing |

*Give information separately for cases and controls in case-control studies and, if applicable, for exposed and unexposed groups in cohort and cross-sectional studies.

**Note:** An Explanation and Elaboration article discusses each checklist item and gives methodological background and published examples of transparent reporting. The STROBE checklist is best used in conjunction with this article (freely available on the Web sites of PLoS Medicine at http://www.plosmedicine.org/, Annals of Internal Medicine at http://www.annals.org/, and Epidemiology at http://www.epidem.com/). Information on the STROBE Initiative is available at www.strobe-statement.org.
